# Identity Diffusion as the Organizing Principle of Borderline Personality Traits in Adolescents—A Non-clinical Study

**DOI:** 10.3389/fpsyt.2021.683288

**Published:** 2021-07-06

**Authors:** Adrienn Rivnyák, Melinda Pohárnok, Bernadette Péley, András Láng

**Affiliations:** Faculty of Humanities and Social Sciences, Institute of Psychology, University of Pécs, Pécs, Hungary

**Keywords:** identity diffusion, borderline personality disorder, adolescence, network analysis, AIDA

## Abstract

Growing evidence shows that diagnosing and treating borderline personality disorder (BPD) is of high relevance for affected youths. Although identity crisis is part of the normative developmental process, identity diffusion is a potential candidate for being an appropriate concept in further developing screening tools and interventions for BPD treatment in adolescence. We hypothesized that severity of borderline traits (as indicated by the strength of their associations with identity diffusion) would be negatively associated with non-clinical adolescents' endorsement of borderline features' presence. We also hypothesized that identity diffusion had a central role in the network of borderline personality traits and could be conceived of as a latent organizing principle of borderline personality disorder. In our study, 169 non-clinical adolescents (81 girls and 88 boys; *M*_age_ = 15.38; SD_age_ = 1.52) filled out self-report measures of borderline personality features and identity diffusion. According to our results, having strong feelings and interpersonal sensitivity were the two most endorsed borderline personality features. Borderline personality features were positively correlated with identity diffusion. The more severe a borderline personality feature was, the less relevant it was for non-clinical adolescents. According to a network analysis, identity diffusion was the most central and least redundant element of the network of borderline personality traits. Results are discussed from a clinical point of view, further encouraging professionals to use identity diffusion screening tools to detect BPD in adolescence.

## Introduction

This paper presents a study that investigated the role of identity diffusion in the organization of borderline personality features in adolescents. The dimensional approach to personality disorders in DSM-5 (1) allows us to make cautious inferences for clinical issues from non-clinical samples as the one in our study. We start by introducing concepts related to normative and pathological identity development in adolescence.

### Identity Development in Adolescence

Both parents and clinicians face adolescence as a challenge. In today's society the developmental stage of adolescence has been prolonged or even to some degree blurred with what we call emerging adulthood (2). Nevertheless, this stage of life—from 10 to 24 years of age for adolescence (3) and from 18 to 25–30 years of age for emerging adulthood (2)—remains one with significant transformations ranging from biological to psychological and social. Although most of adolescents and their families report a trouble-free transition from childhood to adulthood, this period has been frequently described as one of “storm and stress” (4, 5). As part of the normative developmental processes, heightened emotionality—especially in relation to social cues—is a hallmark for adolescent transformation [for a psychopathology related summary see (6)]. Turbulences are also caused by a normative maladaptive shift in emotion regulation including rumination and aggression (7). Thus, normative changes in adolescence might seem to be similar to borderline traits (for a detailed elaboration of this issue see section Borderline personality disorder in adolescence).

The potential turbulences of this developmental stage are not surprising, given the several profound tasks that have to be solved in order to achieve psychologically balanced adult functioning; no matter whether at the end of adolescence or emerging adulthood [for a list of tasks see (8)]. These tasks can be summarized under the identity achievement vs. role confusion psychosocial developmental stage of Erikson (9, 10). Erikson [(11), p. 94] defines ego identity as “the accrued confidence that one's ability to maintain inner sameness and continuity. is matched by the sameness and continuity of one's meaning for others.” Relying on this definition and a review of social-cognitive and psychopathology oriented psychodynamic accounts of identity, Goth et al. (12) suggest two meaningful components of identity development: continuity and coherence. Both components are represented in three domains of psychosocial functioning: intrapersonal, interpersonal, and the level of mental representations.

On the one hand, continuity is the vital experience of subjective self-sameness with an inner stable timeline. Continuity is reflected in the three different domains of psychosocial functioning as goals, talents, commitments, roles, and relationships, and an ability to trust and rely on emotions. On the other hand, coherence is reflected as consistency in self-representations, autonomous psychological functioning with sufficient ego strength, and differentiated mental representations of self and others. By definition, coherence can be considered as the relatively contradiction-free and reflected content of self-representations.

For Erikson (9, 13), it was pivotal to make a distinction between normative identity crisis and identity diffusion. The source of normative identity crisis is development itself. By adolescence, childhood introjections and identifications lose their adaptive function, thereby forcing adolescents to revise them and integrate them into their ego identity at a more abstract level. Thus, identity crisis is a universal component of adolescent psychosocial development. Contrastingly, identity diffusion is the failure to solve the crisis successfully and falling short of achieving a continuous and coherent identity. For Kernberg (14, 15), identity diffusion results from the adolescent's inability to solve the ambivalence of newly achieved independence and attachment to parents and to integrate mental representations of self and others.

### Borderline Personality Disorder in Adolescence

Borderline personality disorder (BPD) is a severe psychiatric disorder with chronic suicidality, unstable interpersonal relationships, and intense and fluctuating emotions (1). Being a very heterogeneous construct, there are 256 unique combinations of the nine diagnostic criteria for BPD. Moreover, factor analytic studies found multiple underlying latent factors explaining BPD criteria. Becker et al. (16) found four factors in a sample of adolescent inpatients. The four factor were (1) “suicidal threats or gestures” and “emptiness or boredom,” (2) “affective instability,” “uncontrolled anger,” and “identity disturbance,” (3) “unstable relationships” and “abandonment fears,” and (4) “impulsiveness” and “identity disturbance.” In a community-based sample, Chabrol et al. (17) found six factors: (1) dissociative/psychotic symptoms, (2) substance use, (3) interpersonal instability, (4) affectivity/identity disturbances, (5) narcissistic features, and (6) impulsivity. In a French-speaking international sample of adolescents diagnosed with BPD, Speranza et al. (18) found two factors accounting for 66.8% of variance in the nine criteria. The two factors were (1) internally oriented and (2) externally oriented criteria, composed of avoidance of abandonment, identity disturbance, chronic feeling of emptiness, and stress-related paranoid ideation for internally oriented criteria and unstable relationships, impulsivity, suicidal or self-mutilating behaviors, and inappropriate anger for externally oriented criteria. From these results we can conclude that albeit there is a single label for this disorder in taxonomy, BPD is a very heterogeneous construct.

Growing evidence shows that BPD is a valid, reliable, and clinically meaningful construct in adolescence (19, 20). The importance of emphasizing and promoting the BDP diagnosis for adolescents is twofold. First, BPD is highly prevalent (every fifth patient in the clinical setting is diagnosed with BPD) and highly dysfunctional (high comorbidity, increased risk for incarceration) mental disorder (21). Second, interventions in adolescence are or should be of high priority because of the malleability and flexibility of this developmental period (22). Successful interventions—even in case of subsyndromal BPD features—can serve as indicated prevention for adult BPD (22).

At the same time, professionals are still hesitant in many settings around the world to diagnose BPD in adolescents (23). The four main reasons for avoiding BPD diagnosis are: (1) invalidity of BPD diagnosis for adolescence, (2) the ongoing process of personality development, (3) difficulty to distinguish normative processes from BPD symptoms, and (4) strong stigmatization [Griffiths (24), Laurenssen et al. (25); for a general review on personality pathology in adolescence see (26)]. The first three of the above mentioned counter-arguments can be rejected based on empirical evidence. As for the validity of BPD diagnosis, prevalence and temporal stability of the diagnosis are very similar in adolescents and adult (27–29). Although personality development is an ongoing process and maturation during adolescence is evident [e.g., (30)], there is also substantial evidence for the stability in adolescence in personality traits (31). The difficulty to make a distinction between normative processes and BPD symptoms can be rejected using a dimensional approach to personality traits and personality disorder symptoms (32). Accordingly, we don't need qualitatively different traits to be present for sine morbo and personality disordered adolescents, a difference in frequency or intensity would suffice. Because stigmatization is highly dependent upon health care professionals' knowledge about BPD (33), progress in the three before mentioned domains could also decrease BPD-related stigmatization.

### Borderline Personality Disorder and Identity Diffusion

Identity—a key process in normative adolescent development—plays an important role in the development and organization of BPD symptoms [e.g., (34, 35)]. The Alternative Model for Personality Disorders (Section III of DSM-5) (1) sees identity disturbance as a central construct in diagnosing personality disorders in general, and especially BPD. Moreover, impairments of identity affect other domains related to personality pathology. Identity diffusion interferes with pursuing goals (self-directedness), understanding others' perspectives (empathy), and establishing close relationships (intimacy) (34). Richtein et al. (35) showed in both clinical and non-clinical samples that together with affective instability, identity diffusion played a central role in the network of BPD symptoms. In a recent review, Kaufman and Meddaoui (36) called for a deeper empirical understanding of identity pathology. Identity diffusion could play a central role in building a unifying theory of BPD, because it is associated with constructs that form the core of BPD in different etiological models [impared mentalizing (37); distorted object relations (14, 15); invalidating environment (38); emotion dysregulation (39)]. Moreover, Wilkinson-Ryan and Westen (40) found that identity diffusion—especially painful incoherence—successfully distinguished patients with BPD from patients with other personality disorders and from individuals with no diagnosis.

### Aims of the Study, Hypothesis

Based on the above presented theoretical background, the aim of the study was twofold. First, we wanted to further evidence that borderline personality features are not to be confused with signs of normative adolescent identity crisis. We hypothesized that severity of borderline traits [as indicated by the strengths of their correlations with identity diffusion—a sign of developmental breakdown (41)] is negatively associated with non-clinical adolescents' endorsement of borderline features' presence. Second, we wanted to test the relevance of identity diffusion in organizing borderline personality features. We hypothesized that identity diffusion had a central role in the network of borderline personality traits and could be conceived of as a latent organizing principle of borderline personality disorder.

## Method

### Sample and Procedure

Our participants were students from secondary schools in Pécs (South-Western Hungary). After parental informed consent 169 adolescents (81 girls and 88 boys) filled out the questionnaire package in paper–pencil format in groups of 20–30. Participants' age was between 12 and 18 with a mean age of 15.38 (SD = 1.52). The study was approved by the Hungarian United Ethical Review Committee for Research in Psychology (Ref. No.: 2017-110).

### Measures

Identity diffusion was measured by Assessment of Identity Development in Adolescence [AIDA; Goth et al. (12); Rivnyák et al. (42) for the Hungarian version]. AIDA is a self-report measure of identity development to differentiate between normative adolescent identity crisis from the clinically relevant state of identity diffusion. The measure consists of 58 items that are evaluated on 5-point Likert-scales based on whether they describe the participant or not. Although AIDA measures different aspects of identity diffusion, we only used the total score in this study. Higher scores refer to more diffuse identity. AIDA proved to be a unidimensional measure of identity diffusion with high internal reliability (Cronbach's α = 0.94).

Borderline personality traits were measured with Borderline Personality Features Scale for Children-11 [BPFSC-11; (43)]. The scale was translated from English into Hungarian using the parallel back-translation procedure (44). The scale consists of 11 items tapping into the main characteristics of borderline personality disorder in the domains of emotional instability, emotional problems, and impaired interpersonal relations. Participants rate their agreement with the statements on 5-point Likert-scales. Higher scores refer to more prominent presence of borderline traits. A Cronbach's α value of 0.79 showed adequate internal reliability of BPFSC-11.

### Statistical Analyses

To describe the variables means and standard deviations were computed. Skewness and kurtosis values were used to describe distribution. Internal reliability of the scales was indicated by Cronbach's α values. Pearson's correlations were used to test the association between variables. The above mentioned statistical analyses were run on IBM SPSS Statistics 22.

To investigate the network of the variables, we used network analysis with JASP 0.9.1.0. To achieve stable and easily interpretable networks, EBICglasso estimation was used. Based on Bayesian parameters and using the Graphical Least Absolute Shrinkage and Selection Operator (GLASSO), this estimation filters out weak correlations and false positive associations resulting from partial correlational analyses.

Networks can be described by several parameters (45–47). Node-related parameters can refer to the centrality of the node (i.e., variable) in the network. Betweenness refers to how many times a node is part of the shortest path between any pair of nodes. Closeness describes how many edges are needed to reach other nodes. Degree refers to how many and how strong edges depart from a node. Higher values refer to the more central role of the node in the network.

Local clustering coefficients quantify how close a node's neighbors are to being a complete graph. Thus, nodes with high local clustering coefficients are redundant in the network. Therefore, lower local clustering coefficients refer to the unique information attributed to a node (i.e., variable). There are several different methods to calculate local clustering coefficients. Costantini et al. (45) suggest to use the coefficient elaborated by Zhang and Horvath [2005 in Costantini et al. (45)] in the case of adaptive LASSO estimations. All centrality and local clustering coefficients reported in this study are standardized values. This means that the value zero refers to a mean value and values 1.0 and −1.0 refer to one standard deviation above and below mean, respectively.

## Results

First, the descriptive characteristics of measured variables are presented. According to the kurtosis and skewness values (Table 1), all reported variables were considered to represent normal distributions (48).

**Table 1:** Descriptive statistics for measured variables.

	**Correlation (*r*) with identity diffusion (AIDA total)**	***M***	**SD**	**Skewness**	**Kurtosis**
Identity diffusion (AIDA total)	n/a	72.61	32.84	0.329	−0.329
Borderline personality traits (BPFSC-11 total)	0.831^*^	25.40	7.39	0.364	−0.148
BPFSC-11 item 1 (feel very lonely)	0.584^*^	1.88	0.97	0.904	0.460
BPFSC-11 item 2 (let people know how much they've hurt me)	0.475^*^	2.94	1.30	0.045	−1.091
BPFSC-11 item 3 (feelings are very strong)	0.144	3.66	1.22	−0.563	−0.667
BPFSC-11 item 4 (something important missing about me)	0.649^*^	2.36	1.27	0.528	−0.866
BPFSC-11 item 5 (careless with things)	0.344^*^	1.96	1.02	0.882	0.084
BPFSC-11 item 6 (people have let me down)	0.535^*^	1.78	1.01	1.298	1.160
BPFSC-11 item 7 (go back and forth between feelings)	0.566^*^	2.37	1.35	0.594	−0.876
BPFSC-11 item 8 (do things without thinking)	0.428^*^	1.88	1.10	1.255	0.923
BPFSC-11 item 9 (people will leave and not come back)	0.601^*^	2.43	1.37	0.533	−1.005
BPFSC-11 item 10 (feel about myself change a lot)	0.518^*^	2.37	1.27	0.437	−0.952
BPFSC-11 item 11 (really mean to each other with friends)	0.348^*^	1.76	1.01	1.463	1.735

*Correlations between identity diffusion and BPFSC-11 (scalewise and itemwise)*.

* *p < 0.001*.

The associations between scales and single items were tested with Pearson's correlations. According to the results (Table 1), identity diffusion was strongly and positively related to borderline personality features in general. Identity diffusion also showed positive correlations with all specific borderline traits (i.e., items of BPFSC-11), except for item 3 (feelings are very strong). The strength of significant correlations ranged from moderate to strong. Identity diffusion was most strongly correlated with item 4 (something important missing about me) and item 9 (people will leave and not come back).

Next, we tested the association between the relative severity of specific borderline personality features and their relative relevance for non-clinical adolescents. To do so, we tested the linear correlation between the mean scores of BPFSC-11 items (as an indicator of the relative relevance of borderline features for non-clinical adolescents) and the strength of their correlations with identity diffusion (as an indicator of the relative severity of specific borderline features). With did this as an analog to computing similarity scores for measuring profile agreement (49). Although the correlation was not significant [*r*_11_ = −0.422; *p* = 0.196] because of the low sample size, there is a moderate negative correlation between relative severity (as indicated by each BPFSC-11 item's correlation with identity diffusion) and relative relevance (as indicated by the mean score of each BPFSC-11 item) of borderline personality traits. This means that non-clinical adolescents reported less agreement with borderline features as borderline features' association with identity diffusion increased.

**Figure 1:**
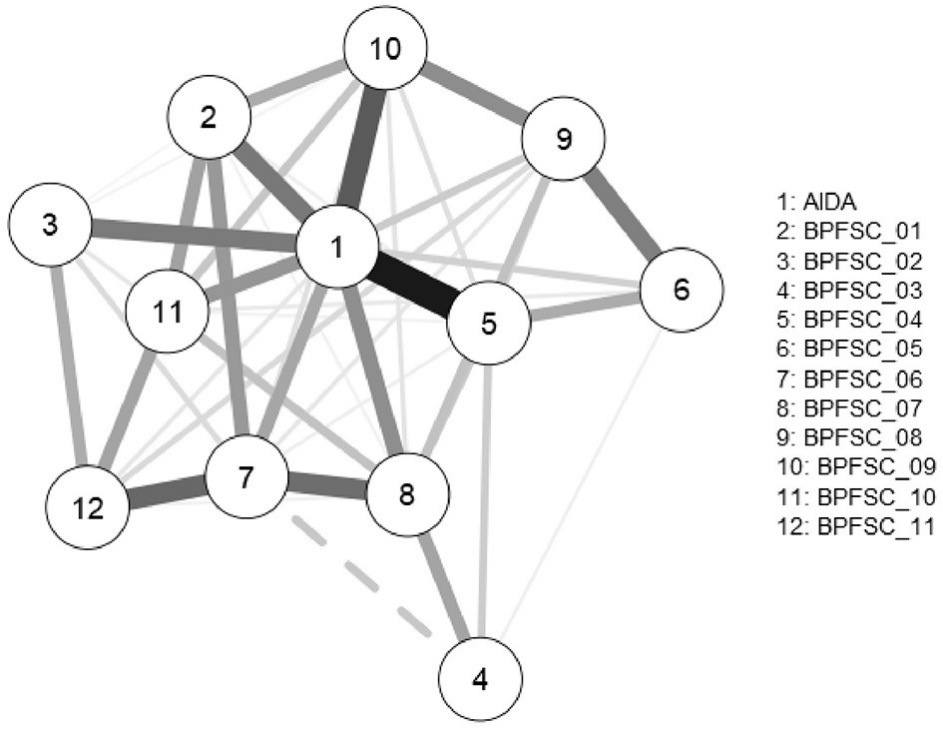
The network of identity diffusion and borderline personality features.

To test the relative importance of identity diffusion in the organization of borderline personality features, we used network analysis. Both visual inspection of the network (Figure 1; see Table 1 for item content) and centrality and local clustering coefficients (Table 2) support the central role of the identity diffusion score in the network. All centrality parameters are the highest for identity diffusion score, while the local clustering coefficient is the lowest for AIDA Total. This means that most of the shortest paths going from a specific borderline trait to another specific borderline trait go through identity diffusion (betweenness), identity diffusion exerts the highest number of direct effects on specific borderline traits (closeness), and identity diffusion has the strongest effect on specific borderline traits (degree). Identity diffusion also has the less redundant (i.e., the most unique) information in the network (local clustering coefficient).

**Table 2:** Characteristic parameters of the network's nodes; centrality and local clustering coefficients (all coefficients are standardized values).

	**Centrality**		**Local clustering**	
	**Betweenness**	**Closeness**	**Degree**	**Zhang coefficient**
Identity diffusion (AIDA total)	2.871	2.187	2.629	−1.413
BPFSC-11 item 1 (feel very lonely)	−0.639	0.251	−0.189	1.548
BPFSC-11 item 2 (let people know how much they've hurt me)	−0.639	−0.151	−0.882	0.947
BPFSC-11 item 3 (feelings are very strong)	−0.639	−1.491	−1.261	0.926
BPFSC-11 item 4 (something important missing about me)	0.271	0.809	0.180	0.247
BPFSC-11 item 5 (careless with things)	−0.639	−1.262	−0.898	0.332
BPFSC-11 item 6 (people have let me down)	0.271	0.215	0.681	−1.034
BPFSC-11 item 7 (go back and forth between feelings)	0.531	0.632	0.378	−0.277
BPFSC-11 item 8 (do things without thinking)	−0.379	−0.728	−0.171	−0.827
BPFSC-11 item 9 (people will leave and not come back)	0.011	0.436	0.094	0.968
BPFSC-11 item 10 (feel about myself change a lot)	−0.639	−0.232	−0.139	−0.037
BPFSC-11 item 11 (really mean to each other with friends)	−0.379	−0.665	−0.423	−1.381

*AIDA, Assessment of Identity Development in Adolescence; BPFSC-11, Borderline Personality Features Scale for Children-11*.

## Discussion

With regard to the first aim of the study (i.e., to investigate the salience of specific borderline personality features in non-clinical adolescents in the conceptual framework of normative adolescent crisis), non-clinical adolescents reported less agreement with more severe borderline personality features (as indicated by the strength of their correlations with identity diffusion). Accordingly, professional concerns about confusing normative identity crisis with borderline personality features [e.g., (50, 51)] might be exaggerated. As adolescents' agreement with BPFSC-11 items increased, single items' strength of correlation with identity diffusion decreased. Thus, our results echo the conclusion of the developers of AIDA (12) and many who make a clear distinction between normative adolescent identity crisis and identity diffusion that is a risk factor for developing borderline personality disorder and personality disorders in general (52, 53).

With regard to the second aim of our study, results of the network analysis supported the hypothesis that identity diffusion could be a latent variable accounting for the interconnectedness of specific borderline personality traits. Although previous factor analytic studies revealed the multi-faceted nature of borderline personality in adolescents (16–18) and Paris (54) even argued that each feature of borderline personality disorder reflects different diatheses, our results showed that identity diffusion—as measured by AIDA (12)—played a central role in the network of borderline personality features in non-clinical adolescents. We suggest that the heterogeneous nature of borderline personality disorder (55) can become less perplexing if the diverse symptoms are conceptualized as stemming from a single source, namely identity diffusion. Nevertheless, we do not question the multiply determined nature of identity diffusion with etiological contributions from genetics to culture (56). In this sense, although distal etiological factor might be diverse, identity diffusion can be hypothesized as a single proximal etiological factor (57).

## Limitations and Conclusions

Although our results are clear and extend previous research in a meaningful way, some limitations of our study should be mentioned. First, the sample size of our study is limited. In order to achieve even stronger conclusions, the sample size should be increased further. Second, although results are compelling, we should be very cautious in extrapolating our conclusions to clinical samples. Therefore, the study should be repeated with a clinical sample.

Our study is among the first to show the central role of identity diffusion as an organizing principle of borderline personality features with network analyses. From a methodological point of view, we join a group of colleagues (45–47) in advocating network analysis as a promising new method in the field of clinical and personality psychology. If clinical studies could replicate our results in the future, they proved identity diffusion to be a potentially useful intervention target in the treatment of adolescents with borderline personality disorder. This would further promote the importance and use of identity diffusion screening tools like AIDA (12).

## Data Availability Statement

The raw data supporting the conclusions of this article will be made available by the authors, without undue reservation.

## Ethics Statement

The studies involving human participants were reviewed and approved by Hungarian United Ethical Review Committee for Research in Psychology (Ref. No.: 2017-110). Written informed consent to participate in this study was provided by the participants' legal guardian/next of kin.

## Author Contributions

AR: study design, data collection, and writing the first draft. MP: writing and revising the first draft. BP: revising the first draft. AL: statistical analysis, writing, and revising the first draft. All authors contributed to the article and approved the submitted version.

## Conflict of Interest

The authors declare that the research was conducted in the absence of any commercial or financial relationships that could be construed as a potential conflict of interest.
